# Adequate antenatal care and ethnicity affect preterm birth in pregnant women living in the tropical rainforest of Suriname

**DOI:** 10.1186/s12884-020-03364-2

**Published:** 2020-11-11

**Authors:** G. K. Baldewsingh, B. C. Jubitana, E. D. van Eer, A. Shankar, A. D. Hindori-Mohangoo, H. H. Covert, L. Shi, M. Y. Lichtveld, C. W. R. Zijlmans

**Affiliations:** 1 Medical Mission Primary Health Care Suriname, Paramaribo, Suriname; 2 Faculty of Medical Sciences, Anton de Kom University of Suriname, Paramaribo, Suriname; 3 Tulane University School of Public Health and Tropical Medicine, New Orleans, USA; 4 Foundation for Perinatal Interventions and Research in Suriname (Perisur), Paramaribo, Suriname; 5 Scientific Research Center Suriname / Academic Hospital Paramaribo, Paramaribo, Suriname

**Keywords:** Antenatal care utilization and content, Birth outcomes, Indigenous, Tribal, Suriname

## Abstract

**Background:**

Adequate antenatal care (ANC) services are key for early identification of pregnancy related risk factors and maintaining women’s health during pregnancy. This study aimed to assess the influence of ANC provided by the Medical Mission Primary Health Care Suriname (MMPHCS) and of ethnicity on adverse birth outcomes in Tribal and Indigenous women living in Suriname’s remote tropical rainforest interior.

**Method:**

From April 2017 to December 2018 eligible Tribal and Indigenous women with a singleton pregnancy that received ANC from MMPHCS were included in the study. Data on low birth weight (LBW < 2500 g), preterm birth (PTB < 37 weeks), low Apgar score (< 7 at 5 min), parity (*≤*1 vs. > 1) and antenatal visits utilization (≥8 vs. < 8) in 15 interior communities were retrospectively analyzed using descriptive statistics, crosstabs and Fisher’s exact tests.

**Results:**

A total of 204 women were included, 100 (49%) were Tribal, mean age was 26 ± 7.2 years and 126 women (62%) had 8 or more ANC visits. One participant had a miscarriage; 22% had adverse birth outcomes: 16 (7.9%) LBW and 30 (14.8%) PTB; 7 women had a child with both PTB and LBW; 5 women had stillbirths. None of the newborns had low Apgar scores. Maternal age, ethnicity, ANC and parity were associated with PTB (χ^2^ = 8,75, *p* = 0.003, χ^2^ = 4,97, *p* = 0.025, χ^2^ = 17,45, *p* < 0.001, χ^2^ = 11,93, *p* < 0.001 respectively).

**Conclusion:**

Despite an almost 100% study adherence over one fifth of women that received ANC in the interior of Suriname had adverse birth outcomes, in particular PTB and LBW. Younger nulliparous Indigenous women with less than the recommended 8 ANC visits had a higher risk for PTB. The rate of adverse birth outcomes highlights the need for further research to better assess factors influencing perinatal outcomes and to put strategies in place to improve perinatal outcomes. Exposure assessment of this sub-cohort and neurodevelopment testing of their children is ongoing and will further inform on potential adverse health effects associated with environmental exposures including heavy metals such as mercury and lead.

## Background

Antenatal care (ANC) services provided by healthcare professionals are key for early identification of pregnancy related risk factors and maintaining women’s health during pregnancy. During 2010–2015, ANC coverage, defined as the percentage of pregnant women aged 15–49 years who attended at least one ANC visit with a skilled health care provider, was approximately 85% globally and 77% in the least developed countries [1]. According to the WHO, an estimated 303,000 women died from pregnancy-related causes worldwide in 2015, while 2.7 million babies died during the first 28 days of life and 2.6 million were stillborn. Both improvement of quality of healthcare during pregnancy and childbirth and increasing the number of ANC visits (≥8) could prevent many of these deaths [1, 2]. In addition, improved prenatal care is also associated with greater maternal satisfaction [3]. Adequate ANC is therefore considered to be effective in reducing complications during pregnancy and delivery [4]. In 2018, the worldwide neonatal mortality rate (NMR) was 18 deaths per 1000 live births, in Latin America and the Caribbean 9/1000 live births. Suriname had the 3rd highest NMR in South America with 10/1000 live births [5].

Suriname is an upper middle income country in northeast South America with an estimated population of 575,991 [6]; 10% of the population lives in the remote tropical rainforest interior. Of the interior population, 83% are Tribal (descendants of runaway African slaves) and 17% are Indigenous (Amerindians). These populations live in relatively small communities with their own cultural beliefs and many speak their own native language. The Medical Mission Primary Health Care Suriname (MMPHCS) is the primary health care provider for the interior population. Annual births in Suriname are estimated at 10,000 [7]. Approximately 93% of all deliveries across the country are facility-based, either in the hospital or at a primary health care clinic [8]. MMPHCS provides ANC as part of its maternal and childcare portfolio and follows the WHO’s recommendations for effective ANC in order to improve maternal and neonatal health outcomes. So far, the quality and effectiveness of ANC provided by MMPHCS for the interior population in Suriname, measured by outcomes such as adverse birth outcomes, has not yet been assessed. In addition, potential differences in birth outcomes related to ANC between Tribal and Indigenous communities with their unique assets, health and cultural traditions were never studied.

This study aimed to evaluate prenatal care, as measured by the quality and number of ANC services provided by MMPHCS on birth outcomes among pregnant women in Suriname’s interior. We hypothesized that adequate prenatal care would have a positively influence on birth outcomes. Also, we assessed potential differences in birth outcomes between Tribal and Indigenous communities.

## Methods

### Antenatal care services

In this prospective cohort study a review of the ANC services was conducted to identify the quality of ANC provided by the MMPHCS. ANC was defined as the total package of care that pregnant women receive from an organized health facility. The three components considered for quality of ANC were: 1) the timing of first ANC visit 2) the number of ANC visits and 3) health services provided during each visit. According to the WHO these three components ensure timely risk identification, prevention and management of pregnancy-related or current diseases and health education and health promotion.

MMPHCS provides primary healthcare services 24/7, including emergency care, maternal and childcare, family planning, management of communicable and non-communicable diseases, school health program, dental care, home visits to elderly and health education and promotion activities. The ANC guidelines include: monitoring of weight, blood pressure and blood sugar, deworming, toxoid immunization, malaria testing and treatment, counseling and testing for Human Immunodeficiency Virus, Syphilis and Hepatitis B, blood and urine testing, iron and folic acid supplementation, uncomplicated delivery at MMPHCS healthcare facility, early identification and referral of high risk pregnancies, health education regarding pregnancy and breastfeeding, and home visits. Adequate ANC services was defined as at least eight ANC contacts with the MMPHCS healthcare provider starting from the first trimester of pregnancy.

There are no specialized neonatal care facilities in the interior. Newborns that need special care are immediately transported to the hospitals in the city. Preferably pregnant women in premature labor are referred in time to the capital city. Reasons for referral to the gynecologist would be risk factors such as grand multiparous women, adolescent nulliparous, hypertension, obesity, anemia or previous C-sections.

### Study population

The study population is a subset of the overall Caribbean Consortium of Environmental and Occupational Health (CCREOH) environmental epidemiologic cohort study that examines the effects of chemical and non-chemical stressors on birth outcomes and pediatric neurodevelopment in mother/child dyads in Suriname. To date 1069 mothers and their infants are included [9].

**Fig. 1:**
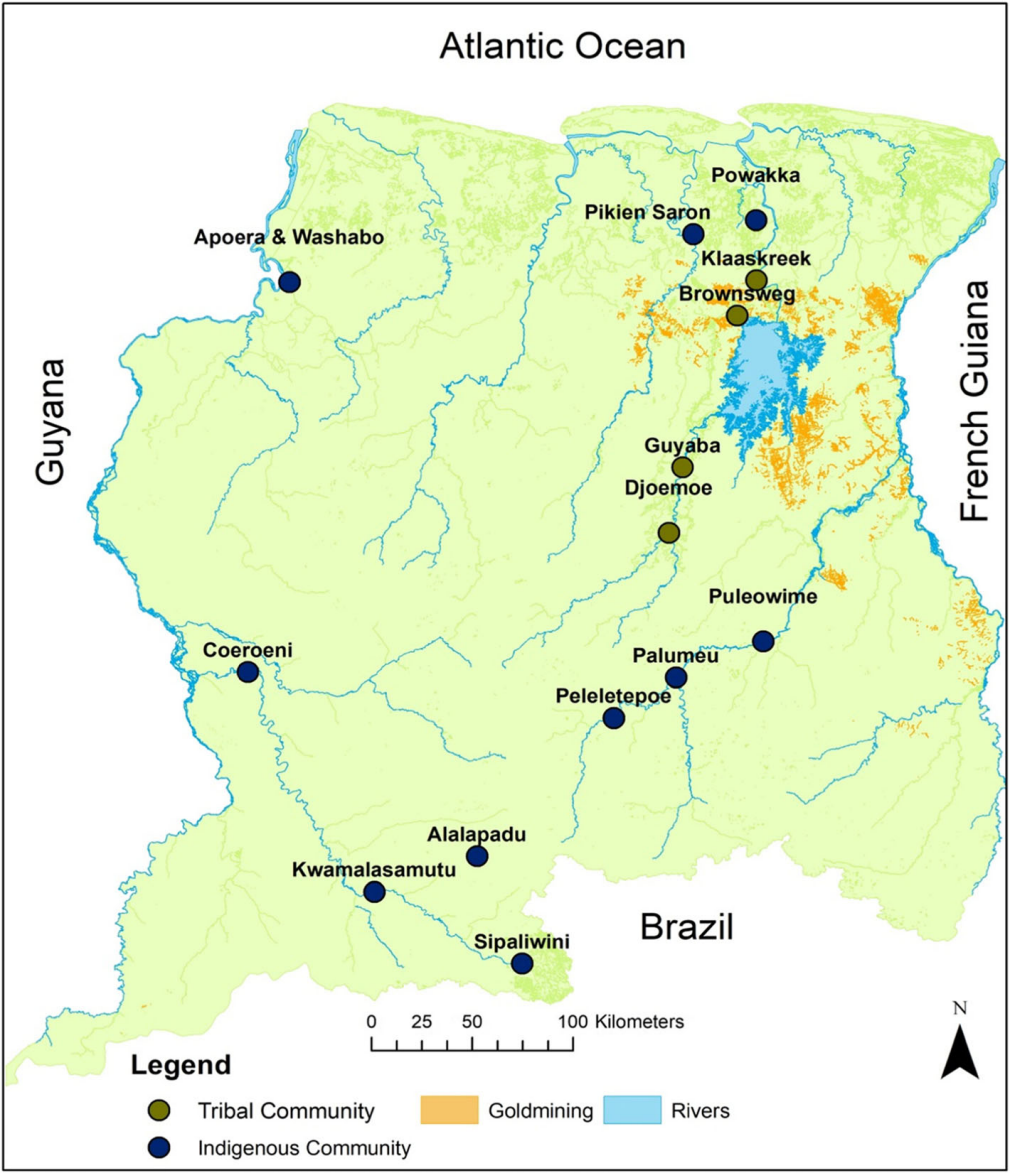
Recruitment areas in the Medical Mission Primary Health Care Suriname regions. Map created in ArcGis 10.1 software; GIS data retrieved from the open source portal www.gonini.org

Women aged 16 years and older with a singleton pregnancy who had registered at one of 15 randomly selected MMPHCS health centers were eligible to enroll in the study (Fig. 1). The interior population consists only of Tribal and Indigenous communities. These are mostly situated far from the capital in remote areas near the large rivers in the interior only reachable by air or water and engage in subsistence livelihood based on local fishing, hunting and growing manioc and other root vegetables. These two communities live in their own regions and hardly interact with each other. We compared those two groups as they are culturally distinct and are of different race and ethnicity.

### Data analysis plan

Descriptive statistics were calculated for the study population and presented as means, standard deviations (SD) for continuous variables and numbers/proportions for categorical variables including age, ethnicity, parity, number of antenatal visits, preterm birth, birth weight, and Apgar score at 5 min.

Categorical variables were assessed using crosstabs. Socio-demographic variables were re-coded for the logistic regression models as follows: age at delivery as < 20 and ≥ 35 vs. 20–34 years; parity as nulliparous or one previous live birth vs. more than one previous live birth; and ethnic group as Tribal vs. Indigenous.

The dependent variables preterm birth (PTB), low birth weight (LBW) and Apgar score (AS) were recoded as binominal variables and defined as follows: PTB as < 37 vs. ≥37 weeks, LBW as < 2500 vs. ≥2500 g, and Apgar score as < 7 vs. ≥7 at 5 min. Gestational age was calculated using the last menstrual period (LMP). Newborns < 28 weeks were excluded from the analyses. Crosstabs were used to explore associations between each of the outcome variables. Differences in proportions between variables were tested with the Pearson’s chi-squared test and if assumptions were not met, Fisher’s exact test was used. Multiple logistic regression was used to develop predictive models for the outcome variables. Variables included in the logistic model were based on the results of the bivariate associations. All analyses were conducted using IBM SPSS version 20 and EPI INFO™ version 7.2.2.6.

### Ethics

Written informed consents in the native languages Saramaccan and Trio were obtained. Ethical approval was granted by the Institutional Review Board of the Government of Suriname (VG 023–14) and the Tulane University Institutional Review Board. Participants were enrolled from April 2017 through December 2018.

## Results

**Fig. 2:**
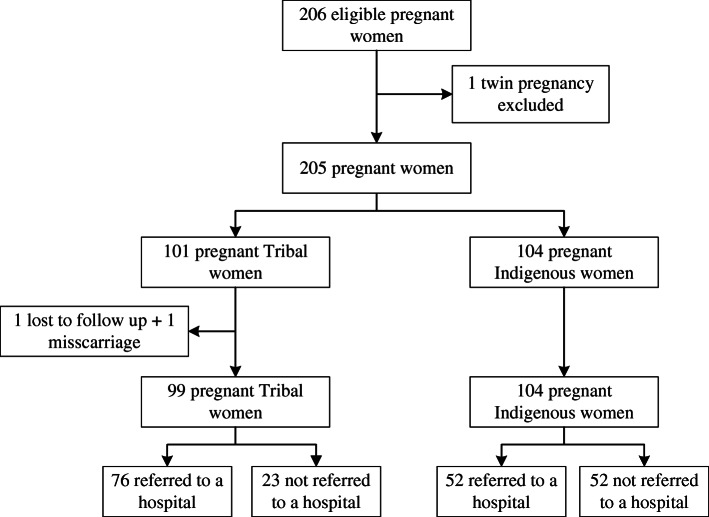
Enrollment flowchart

Of the 206 eligible pregnant women, 203 were ultimately included in the study (Fig. 2). One participant delivered twins and was therefore excluded and one participant from a Tribal community was lost to follow up. 100 (49%) women were from a Tribal community and 104 (51%) were Indigenous. 90% of women returned for follow up visits until the end of their pregnancy. 97% of women had a live birth, 22% had adverse birth outcomes (data not shown). 7 women delivered before 33 weeks gestation, their newborns were excluded from the study as this was one of the exclusion criteria.

The overall mean age (SD) of the participants was 26 years (7.2); 46 (22.5%) were <  20 years of age of which 16 (34.8%) were from a Tribal community and 30 (65.2%) were Indigenous (Table 1). 50% of the Indigenous population in the age group of < 20 years had less than eight ANC visits.

**Table 1:** Characteristics pregnant women receiving antenatal care in the interior of Suriname (*N* = 204)

t1.1	Variable	***N*** = 204	(%)	Mean ± SD	N (%)	Mean ± SD	N (%)	Mean ± SD	***p***-value
t1.2	**Age (years)**				**Tribal (*N*** **= 100)**		**Indigenous(*N*** **= 104)**		
t1.3	Mean			26 ± 7.24		28 ± 7.37		25 ± 6.85	
t1.4	< 20	46	22.5		16 (16)		30 (28.8)		***p*** **= 0.041**
t1.5	20–34	125	61.3		62 (62)		63 (60.6)		
t1.6	≥ 35	33	16.2		22 (22)		11 (10.6)		
t1.7	**ANC no. of visits**								
t1.8	Mean			7.9 ± 2.2		8 ± 2.3		7.6 ± 2.1	
t1.9	< 8	60	29.4		24 (24)		36 (34.6)		*p* = 0.103
t1.10	≥ 8	126	61.8		65 (65)		61 (58.7)		
t1.11	Missing	18	8.8		11 (11.0)		7 (6.7)		
t1.12	**Parity**								
t1.13	Mean			2.7 ± 2.2		3 ± 2.5		2 ± 1.8	
t1.14	< 1	38	18.6		14 (14.0)		24 (23.0)		*p* = 0.178
t1.15	≥ 1	163	79.7		84 (84.0)		79 (76.0)		
t1.16	Missing	3	1.5		2 (2.0)		1 (1.0)		

ANC Antenatal care, *SD* Standard deviation.

116 (72%) participants’ first ANC visit was within the first 4 months of pregnancy. 201 (99%) deliveries were facility-based with a skilled birth attendant present; two women delivered at home. 126 (61.8%) women had eight or more ANC visits, data on ANC visits were missing for 17 (8.3%) of cases. 60% of the Indigenous women had less than 8 ANC visits compared to 48% of the Tribal women (Table 1).

77 (38%) of the women delivered at one of the health facilities in the interior, 127 (62%) were referred to the hospital for delivery. 76 (59%) of the referred participants were of the Tribal and 52 (41%) of the Indigenous community. Reasons for referral for Tribal and Indigenous women were respectively: teenage pregnancy and nulliparity 11 (14.5%) vs. 16 (30.8%), grand multiparity 25 (37.9%) vs. 7 (11.5%) and other (obstructed labor, previous C-section, anemia, hypertension, obesitas) 40 (31.2%) vs. 29 (22.7%).

## Maternal outcomes

10 (4.9%) participants from the Tribal community had pregnancy-induced hypertension of which 3 women developed pre-eclampsia. One participant in the Tribal community had diabetes gravidarum. None of the Indigenous women had either hypertension or diabetes. 187 (92%) of the participants had vaginal delivery. A total of 198 (97%) women had live births, five participants had a stillbirth (3 Tribal and 2 Indigenous), and one Tribal participant had a miscarriage.

The distribution of birth outcomes is presented in Table 2. Of all births, 30 (14.8%) were PTB and 16 (7.9%) had LBW. 7 women had a child with both PTB and LBW. 27.9% (29/104) of the Indigenous study population had either PTB or LBW, while 17% (17/99) of the Tribal had either PTB or LBW. 26 (56.5%) of women with adverse birth outcomes were referred to the capital. There were no differences in PTB and LBW between referred and non-referred Tribal or Indigenous women. None of the newborns had an Apgar score < 7 at 5 min. An Apgar score was missing in 2% (4/198) of the cases.

**Table 2:** Birth outcomes of pregnant women receiving antenatal care in the interior of Suriname (*N* = 203)

t2.1	Variable	***N*** = 203	(%)	Mean ± SD					***p***-value
t2.2	**Birth weight (grams)**				**Tribal (*N*** **= 99)**		**Indigenous (*N*** **= 104)**		
t2.3	Mean			3080 ± 468		3093 ± 515.3		3068 ± 420.7	
t2.4	< 2500	16	7.9		8 (50.0)		8 (50.0)		*p* = 0.385
t2.5	≥ 2500	182	89.6		88 (48.4)		94 (51.6)		
t2.6	Missing	5	2.5						
t2.7	**Preterm birth (weeks)**								
t2.8	Mean			38.6 ± 2.1		39 ± 1.7		38 ± 2.1	
t2.9	< 37	30	14.8		9 (30.0)		21 (70.0)		*p* = 0.046*
t2.1	≥ 37	167	82.2		87 (52.1)		80 (47.9)		
t2.1	Missing	6	3.0						
t2.1	**Apgar score**								
t2.1	< 7 at 5 min	5	2.5						
t2.1	≥7 at 5 min	194	95.5						
t2.1	Missing	4	2.0						
t2.1	**Stillbirth**	5	2.5		3		2		

*Fishers’s exact test

Maternal characteristics associated with LBW and PTB are presented in Table 3. No significant association was observed for age, ethnicity, ANC, and parity and LBW. Age, ethnicity, ANC and parity were associated with PTB, Pearson’s chi-squared χ^2^ = 11,83, *p* = 0.005, χ^2^ = 4,97, *p* = 0.026, χ^2^ = 17,45, *p* < 0.001, χ^2^ = 11,47, *p* < 0.001 respectively. Multivariate analysis for place of birth as dependent and PTB, age, ANC, ethnicity, parity and BW as independent variable showed an association for ANC (< 8 visits, *p* < 0.005) and ethnicity (Tribal, *p* < 0.005) for women who were referred to the hospital.

**Table 3:** Maternal determinants associated with LBW and PTB (*N* = 203)

t3.1		Birth weight				Gestational age			
t3.2		< 2500 g	≥ 2500 g	*p*-value	OR(95%CI)	< 37wks	≥ 37wks	*p*-value	OR(95%CI)
t3.3	**Age**	N (%)	N (%)			N (%)	N (%)		
t3.4	< 20 yr	4 (8.7)	42 (91.3)	0.416*		14 (31.1)	31 (68.9)	0.005	
t3.5	20–34	8 (6.6)	114(93.4)			14 (11.5)	108 (88.5)		
t3.6	≥ 35 yr	4 (13.3)	26 (86.7)			2 (6.7)	28 (93.3)		
t3.7	**Ethnicity**								
t3.8	Tribal	8 (8.3)	87 (91.7)	0.899	1.07 (0.38–2.97)	9 (9.4)	87 (90.6)	0.026	0.39(0.17–0.91)
t3.9	Indigenous	8 (7.8)	94 (92.2)			21 (20.8)	80 (79.2)		
t3.10	**ANC visits**								
t3.11	< 8	2(3.6)	54 (96.4)	0.350*		18 (32.1)	38 (67.9)	< 0.001	5.49(2.33–12.93)
t3.12	≥ 8	11(8.7)	115 (91.3)			10 (7.9)	116 (92.1)		
t3.13	**Parity**								
t3.14	≤ 1	8 (10.8)	66 (89.2)	0.195	1.99 (0.69–5.74)	19 (26.0)	54 (74.0)	< 0.001	3.94(1.72–9.05)
t3.15	> 1	7 (5.7)	115 (94.3)			10 (8.2)	112(91.8)		

*ANC* Antenatal care, *OR* Odds ratio; * Fisher’s exact test

## Discussion

We aimed to assess the utilization of antenatal care and its effect on birth outcomes, specifically PTB and LBW, and potential differences between Tribal and Indigenous pregnant women from Suriname’s interior receiving ANC services from the MMPHCS. ANC adherence of the women enrolled in the study was appropriately high: nine out of ten women returned for follow up visits until the end of their pregnancy. 97% of women had a live birth, 62% were referred to the hospital, 22% had adverse birth outcomes of which 56.5% in the referral group. PTB was positively associated with less number of ANC visits, young age, Indigenous ethnicity, and nulliparity. LBW was not associated with any of these risk factors.

Despite the high ANC adherence, the 14.8% PTB rate observed in this study population was higher compared to the rate of 12% in lower income countries and the 9% rate in high-income countries [10] and was comparable to the country estimate (14%; Verscheuren, et al., Childbirth outcomes and Ethnic disparities in Suriname, accepted for publication April 2020). PTB rate may be influenced by several factors such as gestational age determination, maternal age, ethnicity, parity and ANC utilization. In our study, PTB was determined with LMP based on the participant’s recall. This can be influenced by irregular menses, misinterpretation of vaginal bleeding in early pregnancy as a menstrual cycle, and individual variation in menstruation length cycle. A comparable Brazilian study suggested that the association between young age and PTB may have a biological basis, or that is was caused because of errors in estimation of LMP by young mothers [11]. Hence our approach could have overestimated the prevalence of PTB [12]. We found associations between PTB and age, ethnicity, parity and ANC: PTB was more prevalent in young women < 20 years and in Indigenous women.

8% of the women in this sub-cohort had a newborn with low birth weight, lower than the incidence of LBW in the total cohort (13.1%; Zijlmans et al., Cohort profile CCREOH MeKiTamara cohort study, BMJ Open, accepted for publication) and the country-wide rate of 14.7% [13], and comparable to most countries in Latin America and the Caribbean (Brazil 8.4%, Venezuela 9.1%, Dominican Republic 11.3%) [13]. LBW prevalence was equally distributed among the Tribal and Indigenous participants. Maternal parity is a well-recognized predictor of infant birth weight, with nulliparous women being at greater risk of a child with LBW [14]. In our study one in three participants were primigravida, yet we found no association between parity and LBW. Lower birth weights among first born infants may be a direct consequence of physiological conditions associated with nulliparity, such as maternal pre-pregnancy weight and weight gain [15], these were not recorded in our study. Approximately 84% of the women in this study of which 65% had eight or more ANC visits, had a term birth with birth weight above 2500 g. Observations from a study conducted in Brazil [16] has shown that at least seven ANC visits were protective in LBW incidence. The greater the number of contacts with a healthcare provider, the greater the change of reducing risk factors or treating pregnancy complications with better results of birth outcomes [2].

Neither maternal age, parity nor ANC were associated with birth weight in our study. Several factors that were not included in the analyses of this sub-cohort study could be of influence such as maternal pre-pregnancy weight and weight gain during pregnancy [17], hypertension, and other well-known pre-existing maternal conditions. Women receiving antenatal care provided by MMPHCS are screened for these conditions and in case of early detection of abnormal findings women may be referred to the capital of Suriname. As a result, timely diagnosis and treatment interventions provided within the MMPHCS integrated primary health care program may help to adequately detect risk pregnancies, especially in these remote communities with its disadvantaged populations. MMPHCS refers approximately 60% of women with risk pregnancies annually [18]. Further studies are needed to assess the influence of risk factors for LBW and other adverse birth outcomes in women living in the interior of Suriname.

Two-thirds of our Indigenous study population was younger than 20 years compared to 35% of the Tribal women. Frequent ANC attendance is particularly important in pregnant adolescents aged 15–19 as complications have been shown to be higher among these teenage girls [6]. In this risk group the mother and her offspring compete for nutrients as both are growing and developing [19]. Likewise, pregnant women 35 years and older are at risk for adverse perinatal birth outcomes due to pre-existing and probably undiagnosed diseases [20, 21] and pregnancy prevalence in this age group is increasing both in developed as developing countries over the last decade [22].

Parity was associated with preterm birth: women with first time pregnancies had a higher risk of preterm labor. Studies on the association between parity and preterm birth are inconclusive in their findings: in a meta-analyses no association was found [23], while others report multiparous women were more likely to deliver preterm [24, 25]. Approximately 60% of the Indigenous group of participants had fewer than 8 ANC visits, indicating that less adherence to antenatal care may influence our PTB prevalence results.

In our study utilization of ANC services were 100% covered for at least one visit during the pregnancy. In 2016, the World Health Organization (WHO) updated the Antenatal Care model with recommendation as follows: ANC should commence within the first 4 months of pregnancy and women should have at least eight ANC visits during the course of an uncomplicated pregnancy [2, 26]. Although the number of eight antenatal care visits as part of the maternal and childcare portfolio provided by the MMPHCS for Tribal and Indigenous pregnant women in the tropical rainforest interior of Suriname meets the recommended WHO standards in terms of frequency of ANC visits, women had a significant rate of adverse birth outcomes. The incidence of less than 8 prenatal visits did vary by community, over half of the Indigenous women had less than 8 prenatal visits, this could be attributed to their way of living as these women are more engage in subsistence livelihood based on growing manioc and other root vegetables. The MMPHCS ANC guidelines cover all five WHO recommended components of ANC from obtaining the history of previous pregnancies to offering a home visit within 1 week after delivery. The authors recommend that improving the dissemination of essential information about ANC through the MMPHCS health care workers is critical to ensure that pregnant women living in the interior maximally benefit from these services. The health work force consists primarily of local villagers who receive a four-year health care training by the MMPHCS under supervision of the Surinamese Ministry of Health. These healthcare workers practice according to standardized guidelines and are supervised by MMPHCS physicians. In addition, community education programs regarding for example how to correctly determine the last menstruation period should be emphasized. In addition, expansion of ANC services is recommended including introduction of ultrasounds for more accurately estimating gestational age.

Women living in remote interior communities in Suriname, where mercury is abundantly used in artisanal and small-scale goldmining activities, are known to have high concentrations of mercury in hair [23]. These high mercury concentrations can largely be attributed to the consumption of mercury contaminated fish [24]. In a recent study we conducted we found no differences in birth outcomes associated with downstream proximity to gold mining [25].

One of the strengths of our study is that all pregnant women in our study population received a standardized package of ANC care which covered all the recommendations indicated by WHO. In addition, the data were abstracted from accurately documented medical records. This is also the first study to evaluate the impact of ANC utilization with an almost 100% adherence and frequency of adverse birth outcomes in the interior of Suriname.

The study included some limitations: we did not assess the quality and content of ANC as this may influence the ANC across the different MMPHCS health facilities; secondly, we did not examine the mother’s history of PTB and LBW; mothers with previous PTB or LBW are more likely to have babies with LBW [27]. Also, gestational age was not calculated by ultrasound but instead obtained using the LMP date, therefore the exact gestational age could not be determined. Finally, in Suriname, national data on characteristics of the pregnant population, pregnancy and delivery are not readily available, for example data on body mass index, hypertension, diabetes, pre-pregnancy weight and gestational vascular disease are all correlated to perinatal outcome hampering assessment of potential relevant risk factors.

## Conclusion

Despite the high attendance of ANC, over one fifth of women living in the interior of Suriname that received antenatal care from the Medical Mission Primary Health Care Suriname had adverse birth outcomes, particularly preterm birth and low birth weight. Younger nulliparous Indigenous women with less than the recommended 8 ANC visits were particularly at risk for PTB. The high rate of adverse birth outcomes highlights the need for further research to better assess the factors influencing perinatal outcomes and also strategies to put in place to improve perinatal outcomes. Exposure assessment of this sub-cohort and neurodevelopment testing of their children is ongoing and will further inform other potential adverse health effects associated with environmental exposures. A client satisfaction survey is recommended to evaluate the ANC services provided by the MMPHCS.

## Data Availability

The datasets generated and/or analyzed during the current study are not publicly available due to ongoing data analysis beyond what is currently included in this study but are available from the corresponding author on reasonable request.

## Abbreviations

ANC: Antenatal Care
AS: Apgar Score
CCREOH: Caribbean Consortium of Environmental and Occupational Health
Hg: Mercury
LBW: Low Birth Weight
LMP: Last Menstrual Period
MMPHCS: Medical Mission Primary Health Care Suriname
NGO: Non-Governmental Organization
NMR: Neonatal Mortality Rate
PTB: Preterm Birth
SD: Standard Deviation
WHO: World Health Organization
